# Bixin Protects Against Kidney Interstitial Fibrosis Through Promoting STAT6 Degradation

**DOI:** 10.3389/fcell.2020.576988

**Published:** 2020-11-17

**Authors:** Jianzhong Li, Youjing Yang, Shuhui Wei, Ling Chen, Lian Xue, Hailin Tian, Shasha Tao

**Affiliations:** ^1^Medical College of Soochow University, Suzhou, China; ^2^Department of Nephrology, The First Affiliated Hospital of Soochow University, Suzhou, China; ^3^School of Public Health, Medical College of Soochow University, Suzhou, China

**Keywords:** kidney fibrosis, bixin, STAT6, SQSTM1(P62), tubular cell

## Abstract

Bixin, a natural carotenoid extracted from the seeds of *Bixa orellana*, has antioxidant and anti-inflammation effects. However, the pharmacological effects and underlying mechanisms of bixin in kidney interstitial fibrosis remain unknown. Partial epithelial-to-mesenchymal transition (EMT) of tubular cells has been linked to renal interstitial fibrosis. Here, we found that in the unilateral ureteral obstruction model, bixin administration could ameliorate kidney interstitial fibrosis. The expression of signal transducer and activator of transcription 6 (STAT6) was dramatically increased in renal tubular cells. Bixin treatment inhibited STAT6 induction. The activation of STAT6 signaling was essential for transforming growth factor β1, fibrotic markers, and EMT-related protein expression in HK2 cells, which was confirmed by using the Stat6^–/–^ mice. Ubiquitination, but not the acetylation level of STAT6, was induced by bixin treatment and promoted the suppression of phosphorylation and stability of STAT6. P62-dependent autophagy might be involved in this process. The study demonstrated that bixin can be exploited therapeutically to alleviate renal interstitial fibrosis by targeting STAT6 signaling deactivation.

## Introduction

Chronic kidney disease is recognized as a public health problem worldwide. Irrespective of the etiology, renal interstitial fibrosis is the common pathological feature leading to end-stage renal failure and is characterized by excessive interstitial ECM deposition accompanied with tubular atrophy (Glassock et al., 2017; Romagnani et al., 2017; Djudjaj and Boor, 2019). Recently, it is widely recognized that partial EMT is involved in the progression of ECM production and renal interstitial fibrosis. In this process, damaged epithelial cells undergo G2/M phase cell cycle arrest with the downregulation of their epithelial features and acquirement of a mesenchymal phenotype, accompanied by profibrotic cytokine upregulation (Grande et al., 2015; Lovisa et al., 2015; Zhou et al., 2017; Yuan et al., 2019). TGFβ1 is a crucial mediator for the process of partial EMT (Gewin, 2018; Hajarnis et al., 2018; Qi and Yang, 2018; Liu et al., 2019; Yuan et al., 2019).

The STAT family of transcription factors is composed of at least seven members, named STAT1, 2, 3, 4, 5a, 5b, and 6, with roles in regulating diverse cellular events, including cell differentiation, survival, and proliferation (Miklossy et al., 2013; Abroun et al., 2015). Frequently, T_H_2 cytokines [interleukin 4 (IL4), IL13] bind to its receptor at the plasma membrane and initiates Janus kinase 3 (JAK3)–mediated phosphorylation of STAT6, which is essential for the induction of IL4/IL13-responsive gene transcription (Takeda et al., 1996; Elo et al., 2010; Goenka and Kaplan, 2011; Van Dyken and Locksley, 2013). Previous studies showed that STAT6, as a transcription factor, was able to bind to promoter region of TGFβ1 gene (Kundu et al., 2016). Yan et al. (2015) and Liang et al. (2017) reported that inhibition of IL4R/JAK3/STAT6 signaling pathway could attenuate kidney interstitial fibrosis by suppressing bone marrow–derived fibroblasts activation. However, the role and the underlying mechanisms of STAT6 in tubular cells in regulating kidney interstitial fibrosis remain unknown. STAT6 can be degraded via ubiquitin–proteasome system and the lysosomal pathway in 293T cells (Gu et al., 2018); in addition, transcriptional activity of STAT6 can be suppressed by the acetylation of STAT6 at Lys383 via the acetyltransferase CBP in macrophages (Yu et al., 2019). SQSTM1(P62) is a key adapter of autophagy, escorting relative ubiquitinated proteins to autolysosomes, and plays a vital role in protein degradation (Nezis and Stenmark, 2012; Katsuragi et al., 2015; Aparicio et al., 2019). These observations suggest the inhibition of STAT6 activity may be a potential therapeutic target for renal fibrosis.

Bixin is a carotenoid isolated from the seeds of *Bixa orellana* (Annatto), and it is approved by the FDA for the application of food and cosmetics industries. Bixin displays numerous important pharmacological activities, including anti-inflammatory, antioxidant, lipid-lowering effects, and so on (Silva et al., 2001; Tao et al., 2015, 2016). Our previous study demonstrated that bixin could protect against lung fibrosis by inhibiting the TGFβ1 signaling (Zhang et al., 2018). Moreover, P62 protein level is upregulated following bixin treatment in ciliated cells (Liu et al., 2020).

We therefore sought to determine if bixin could be a potential therapeutic agent to alleviate kidney interstitial fibrosis. The UUO model was used for our fibrosis experiments. We found that STAT6 was highly expressed in the tubular epithelium after UUO, and it was involved in the process of partial EMT and ECM protein expression through *in vivo* and *in vitro* studies. Bixin could alleviate renal interstitial fibrosis by facilitating STAT6 degradation.

## Materials and Methods

### Chemicals, Antibodies, and Cell Culture

Bixin (BI175) was purchased from Spectrum (New Brunswick, NJ, United States). Recombinant human IL4 protein (204-IL-010), recombinant human IL13 protein (214-ILB-005), and recombinant human TGFβ1 protein (240-B-002) were purchased from R&D Systems (Minneapolis, MN, United States). CHX (239763-M), chloroquine (CHQ; C6628), and bafilomycin A1 (BafA1; 19-148) were purchased from Sigma–Aldrich (St. Louis, MO, United States). MG132 (EY0002) was purchased from Amquar (Colorado, United States). Bortezomib (B-1408) was purchased from LC laboratories (Woburn, MA, United States). Primary antibodies against STAT6 (sc-374021), p-STAT6 (sc-136019), FN (sc-18827), α-SMA (sc-53142), Col4A6 (sc-398655), P62 (sc-28359), Ub (sc-8017), CBP (sc-32244), c-myc (sc-40), E-cadherin (sc-8426), N-cadherin (sc-59987), TGFβ1 (sc-130348), and GAPDH (sc-32233) were purchased from Santa Cruz (Shanghai, China). Primary antibodies against Flag (#14793) and acetylated-lysine (#9441S) were purchased from Cell Signaling (Danvers, MA, United States). Antihemagglutinin (HA) epitope antibody was purchased from Covance (Branford, CT, United States). HRP–conjugated secondary antibodies were purchased from Immunoway (Plano, TX, United States; anti-mouse:RS0001, anti-rabbit:RS0002). Alexa Fluor 488 anti-mouse and Alexa Fluor 594 anti-rabbit were purchased from Santa Cruz. Human renal tubular epithelial cell line HK2 was purchased from ATCC (Manassas, VA, United States) were cultured in Dulbecco modified eagle medium (DMEM) supplemented with 10% fetal bovine serum (FBS) (Hyclone, Logan, UT, United States), 100 U/mL penicillin, and 100 μg/mL streptomycin (Invitrogen, Carlsbad, CA, United States). The cells were maintained at 37°C in a humidified incubator containing 5% CO_2_.

### Transfection of Small Interfering RNA and cDNA

cDNA transfection was performed with Lipofectamine 2000 (Invitrogen, Shanghai, China; 11668027) and Hiperfect transfection reagent (Qiagen, Hilden, Germany; 301702) was employed for transfection of siRNA according to the manufacturer’s instructions. Non-targeted siRNA (Ctrl siRNA, #1027281) and P62-targeted siRNA (P62 siRNA #SI00057596) were purchased from Qiagen. STAT6-targeted siRNA (STAT6 siRNA) was purchased from GenePharma, Shanghai. For siRNA transfection, 3 × 10^5^ cells per well were seeded in 6-well plate and a mixture containing 300 ng of the indicated siRNA along with 12 μL Hiperfect transfection reagent was added into the cells for the indicated siRNA transfection at the same time. For cDNA transfection, 4 × 10^5^ cells per well were seeded in 6-well plate for 24 h. The mixture of 1 μg cDNA and 3 μL Lipofectamine 2,000 diluted in serum free medium was added into the cells for 6-h incubation. After adding the fresh serum free medium, the cells were used for the subsequent experiments.

### Cell Viability Detection

The toxicity of bixin in the HK2 cells was measured by functional impairment of the mitochondria using 3-(4,5-dime thylthiazol-2-yl)-2,5-diphenyltetrazolium bromide (MTT from Sigma-Aldrich). Approximately 1 × 10^4^ cells per well were seeded in a 96-well plate. After 24-h incubation, the cells were treated with multiple doses of bixin for 48 h. Then 40 μg MTT was added into the cells. After 2-h incubation, the medium was removed, and 100 μL isopropanol/HCl was added into each well to dissolve the crystals. Absorbance at 570 nm was measured using a Synergy 2 Multi-Mode Microplate Reader (Biotek, Seattle, United States).

### Immunoblot Analysis, Immunoprecipitation, Ubiquitylation Assay, Protein Half-Life Assay, Indirect Immunofluorescence, and Live-Cell Imaging

The immunoblot analyses were employed to detect the protein expression. Cell and tissue lysates were prepared the same as previously reported (Tao et al., 2013). Lysates were resolved by sodium dodecyl sulfate–polyacrylamide gel electrophoresis and transferred onto a nitrocellulose membrane for immunoblot analyses with the indicated antibodies. For immunoprecipitation and the ubiquitination assay, cells were harvested in RIPA buffer (Thermo) and incubated with 1 μg anti-STAT6 antibody with protein A agarose beads (Invitrogen) or HA-conjugated magnetic beads (Bimaker) at 4°C for 16 h. Immunoprecipitated proteins were analyzed by immunoblot with antibodies against Ub, p-STAT6, HA, and acetylated-lysine. To clarify STAT6 stability, cell lysates at different time points from control or bixin-treated cells were subjected to immunoblot analyses with the anti-STAT6 and anti-GAPDH antibodies. The intensity of STAT6 and GAPDH bands was quantified with ImageJ and plotted against the time after the addition of CHX. For indirect immunofluorescence, cells were seeded on round glass coverslips (Fisher Scientific). After fixing with chilled methanol for 15 min, coverslips were incubated with the primary antibodies and the respective secondary antibodies for 50 min each. Then the coverslips were mounted with antifade mounting solution (Invitrogen). For live-cell imaging assay, cells were passaged on 35-mm glass-bottom dishes (*In Vivo* Scientific). After washing with phosphate-buffered saline, cells were imaged in phenol red free DMEM with 10% FBS. Images were captured with a fluorescence microscope (Leica DM 2500).

### RNA Extraction and Quantitative Real-Time Polymerase Chain Reaction

Total RNA was extracted from HK2 cells and kidney tissues with TRIzol reagent purchased from CWBIO (Beijing, China). cDNA was acquired with equal amounts of RNA using HiFiScript cDNA synthesis kit according to the manufacturer’s instructions (CWBIO, Beijing, China). Primer sequences of human P62, FN, TGFβ1, and GAPDH were described previously (Zhang et al., 2018), and the primer sequences of human Col4A6 and STAT6 were as follows: STAT6-F:GTCTGGTCTCCAAGATGCCC; STAT6-R:ATATGCTCTCAAGGGTGCTGA. Col4A6-F:CTCC TTGCCCTCACTCATAGC; Col4A6-R:GTCTCCCTTAGGCC CTTTAGG.

The real-time polymerase chain reaction (RT-PCR) conditions used were: initial denaturation (95°C, 10 min), 40 cycles of amplification (95°C, 15 s; 60°C, 1 min), melting curve (95°C, 15 s; 60°C, 1 min; 95°C, 15 s; 60°C, 15 s). Mean crossing point (*C*p) values and SDs were determined. *C*p values were normalized to the respective *C*p values of human GAPDH reference gene. Data are presented as a fold change in gene expression compared to the control group.

### Animals and Treatments

Stat6 wild-type (Stat6^+/+^) and Stat6 knockout (Stat6^–/–^) mice were obtained by breeding Stat6 heterozygous mice. C57BL/6 mice (7 weeks old) were obtained from SLAC Laboratory (Shanghai, China). All mice were maintained in 12 h/12 h light–dark cycle, pathogen-free condition with water and food *ad libitum*. Eight-week-old mice were used for this study. Stat6^+/+^ and Stat6^–/–^ mice were randomly divided into four groups (*n* = 6 per group): control group (Ctrl), bixin-treated group (bixin), unilateral ureteric obstruction group (UUO), bixin-treated UUO group (UUO + bixin). Bixin (100 mg/kg) was i.p. injected 1 day before UUO and administered once every 3 days. To induce renal fibrosis, the UUO procedure was performed as described (Li et al., 2015). Briefly, mice were anesthetized with chloral hydrate (10 mL/kg; Sigma-Aldrich). The right ureter was exposed and completely ligated using fine suture material (4–0 silk) at two points with a flank incision. The study protocols followed the Guide for the Care and Use of Laboratory Animals and were approved by the Soochow University Institutional Animal Care and Use Committee.

### Hematoxylin and Eosin, Immunohistochemistry, and Masson Staining

Renal tissues were dehydrated, cleared, infiltrated by paraffin wax, and sequentially embedded in the paraffin followed by fixing with 4% paraformaldehyde for 24 h, and the slides (4 μm) were cut. H&E staining was used to assess the morphologies of tissues with the indicated treatment. The procedures of IHC analysis were performed as previously described (Xue et al., 2018). Positive protein’s staining was performed by EnVision + System-HRP kit (Dako) based on the manufacturer’s instructions. Masson staining was performed by Masson Trichrome Stain Kit (Yuanye, Shanghai) according to the manufacturer’s instructions. The images were captured and analyzed by a fluorescence microscope (Leica DM 2500).

### Statistics

The results were presented as means ± SD. SPSS 19.0 was used to perform the statistical tests. Student *t* test (unpaired) was employed to compare multiple groups, whereas one-way analysis of variance with Bonferroni correction was used to adjust the means of three or more groups. *p* < 0.05 was considered to be statistically significant.

## Results

### Bixin Prevents the Renal Interstitial Fibrosis Caused by UUO

**FIGURE 1:**
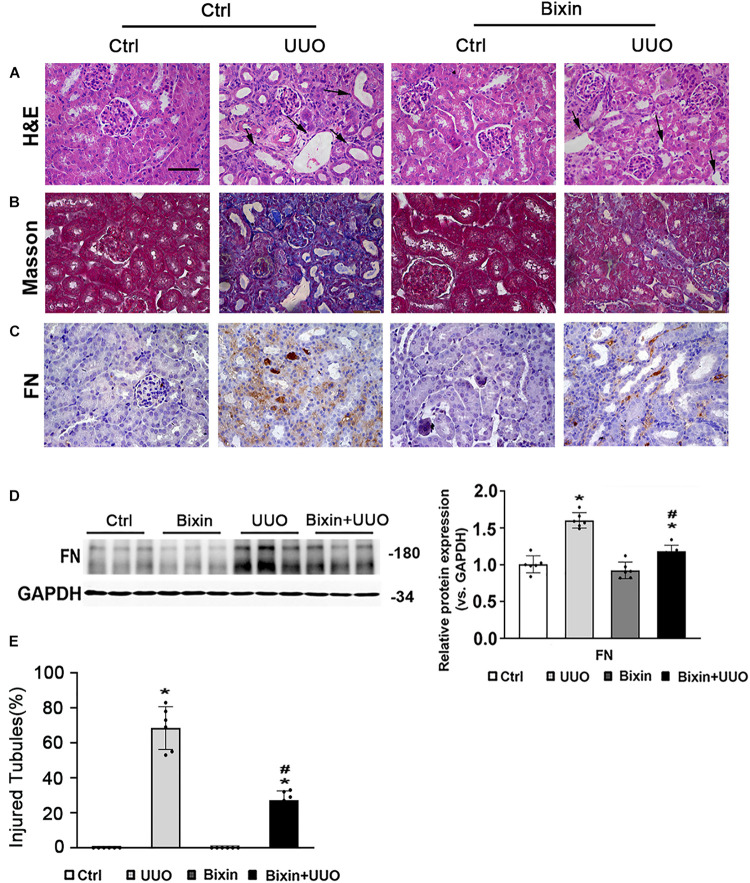
Bixin prevents the renal interstitial fibrosis caused by UUO. Kidney sections from the indicated group of mice were subjected to H&E **(A)** (black arrows: dilated and atrophic tubules), Masson **(B)**, and IHC staining of FN **(C)**; the representative images are shown; scale bar = 50 μm. **(D)** Kidney tissue lysates from each group were subjected to immunoblot analyses with the FN. Representative blots of three independent samples in each group were shown; quantification of relative protein expression was determined, results are expression as means ± SD (*n* = 6; *p < 0.05, Ctrl vs. UUO group; ^#^*p* < 0.05, UUO group vs. bixin + UUO group). **(E)** The graph showing the percent of injured tubules for kidney tissues within groups (*n* = 6; *p < 0.05, Ctrl vs. UUO group; ^#^*p* < 0.05, UUO group vs. bixin + UUO group).

To examine the role of bixin on the renal interstitial fibrosis, C57BL/6 mice were subjected to UUO for 7 days. Bixin was i.p. injected 1 day before UUO treatment and once every 3 days accordingly. Kidney sections from the different groups were fixed and stained for the assessment of pathological changes and ECM deposition. As shown by H&E staining, UUO induced remarkable renal damage with numbers of dilated and atrophic tubules and obvious inflammatory cell infiltration in control group mice, which was attenuated in bixin-treated UUO mice (Figures 1A,E). Masson and FN staining showed that UUO caused a huge ECM deposition in renal tissues of control group mice, whereas less ECM was detected in the kidney of bixin-treated UUO mice (Figures 1B,C). Besides that, the tissue lysates were also subjected to the immunoblot analysis. The results indicated that bixin decreased the induced level of FN in the kidney of the mice with UUO (Figure 1D). Taken together, these results indicated that bixin could prevent the renal fibrosis caused by UUO.

### Bixin Inhibits the Upregulation of STAT6 and ECM Protein Expression in Tubular Cells Under the Profibrogenic Environment

**FIGURE 2:**
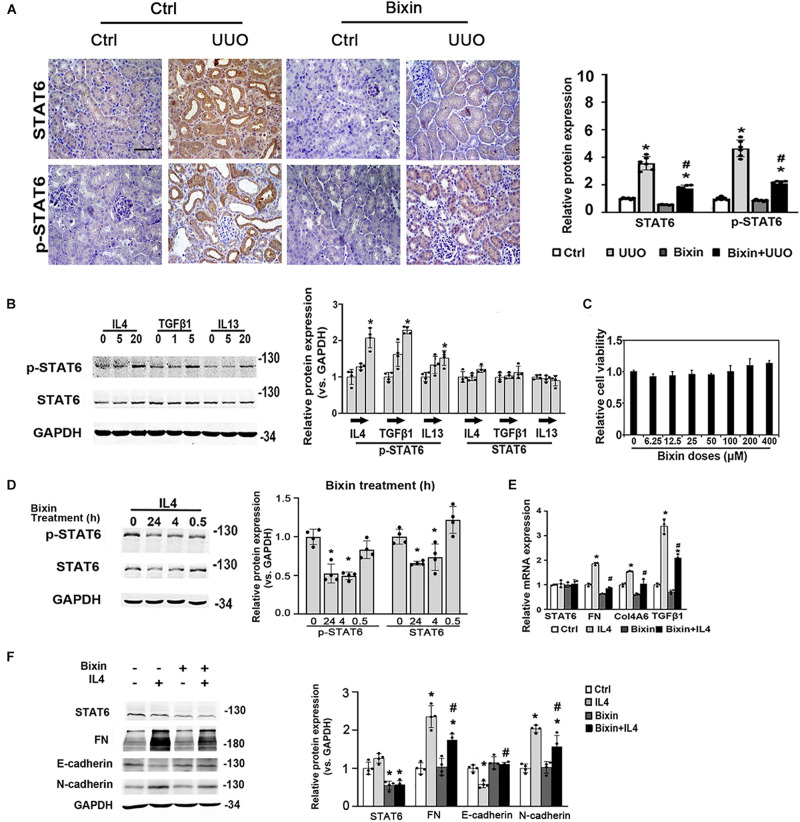
Bixin inhibits the upregulation of STAT6 and ECM protein expression in tubular cells under the profibrogenic environment. **(A)** Kidney sections from the indicated group of mice were subjected to STAT6 and p-STAT6; the representative images are shown; scale bar = 50 μm. Quantitative analysis of the indicated protein expression in the kidney was determined; results are expression as means ± SD (*p < 0.05, Ctrl vs. UUO group; ^#^*p* < 0.05, UUO group vs. bixin + UUO group; *n* = 6). **(B)** Followed by 24-h serum free medium culture, HK2 cells were treated with IL4, TGFβ1, and IL13 with the indicated doses for 24 h. Cells were lysed and subjected to the immunoblot analysis with the indicated antibodies; quantification of the relative protein expression was determined. Results are expressed as means ± SD (*p < 0.05, Ctrl vs. treatments; *n* = 4). **(C)** Cell viability was measured in HK2 cells with the indicated doses of bixin treatment for 48 h. **(D)** After 24-h serum free medium culture, HK2 cells were pretreated with IL4 (20 ng/mL) for 24 h and separately administered with the bixin (40 μM) for the different time points. Cells were lysed and subjected to the immunoblot analysis with the indicated antibodies; quantification of the relative protein expression was determined. Results are expressed as means ± SD (*p < 0.05, Ctrl vs. bixin treatments; *n* = 4). **(E)** HK2 were starved with serum free medium for 24 h and pretreated with bixin (40 μM) for 24 h. Then, treated with IL4 (20 ng/mL) for another 24 h, the cells were harvested, and total RNA was extracted. mRNA levels of STAT6, FN, Col4A6, and TGFβ1 were measured by qRT-PCR assay. Results are expressed as means ± SD (*p < 0.05, Ctrl vs. treatments; ^#^*p* < 0.05, IL4 treatment vs. bixin + IL4 treatment; *n* = 3). **(F)** HK2 cells were treated with bixin (40 μM) and/or IL4 (20 ng/mL) for 24 h. Cells were harvested, and the lysates were subjected to immunoblot analyses with the antibodies against STAT6, FN, E-cadherin, N-cadherin, and GAPDH; quantification of the relative protein expression was determined. Results are expressed as means ± SD (*p < 0.05, Ctrl vs. treatments; ^#^*p* < 0.05, IL4 treatment vs. bixin + IL4 treatment; *n* = 4).

To determine whether STAT6 signaling is involved in the protective beneficial effect of bixin on kidney fibrosis, here the expression of STAT6 was first investigated in the kidneys harvested 7 days after UUO. The results showed that there was a dramatic increase of STAT6 and p-STAT6 expression in the kidneys, especially in the renal tubular epithelium (Figure 2A). To further detect the activation state of the STAT6 pathway in tubular cells in response to profibrotic stimuli, a renal tubular epithelial cell line (HK2) was employed. As expected, the phosphorylated levels of STAT6 (p-STAT6) were upregulated by type II cytokines (IL4 and IL13) at 24 h. In addition, the expression of STAT6 was not changed. p-STAT6 could also be upregulated as well after the cells were treated with TGFβ1 (Figure 2B), which indicated that the activation of STAT6 in the tubular epithelium might be involved in the pathological changes induced by UUO. To explore the effects of bixin on the STAT6 expression in tubular cells, we first employed an MTT assay to assess the cytotoxicity of bixin in HK2 cells. The data showed that there was no observed toxicity at the doses below 400 μM (Figure 2C). Combined with our previous study, 40 μM was picked up for the subsequent experiments. Then the mediation of p-STAT6 and STAT6 by bixin was determined. As shown in Figure 2D, bixin decreased the protein expression of p-STAT6 and STAT6 on the IL4-induced levels. Moreover, in the kidneys subjected with UUO, bixin treatment largely suppressed p-STAT6 and STAT6 expression in the tubular cells compared with the control group (Figure 2A). To evaluate whether bixin could function as the prevention of ECM-related protein expression *in vitro*, HK2 cells were treated with bixin and/or IL4 for 24 h. The results from quantitative RT (qRT)–PCR assay showed that IL4 treatment induced the mRNA expression of FN and Col4A6, which was suppressed by bixin treatment (Figure 2E). In addition, TGFβ1, as the target of STAT6 signaling, was also downregulated by bixin as well. Immunoblot analysis confirmed the results that bixin could attenuate IL4 caused the dysregulation of FN expression (Figure 2F). According to the previous studies, the partial EMT of renal tubular epithelium is a critical cause of interstitial fibrosis, which was mainly regulated by TGFβ1 signaling (Yuan et al., 2019). Then we also detected the EMT-related proteins, the epithelium biomarker E-cadherin was decreased by the administration of IL4, which could be reversed by bixin treatment. The mesenchyme biomarker N-cadherin was increased with the IL4 treatment, and attenuated by bixin treatment as well (Figure 2F). Collectively, these results suggest that STAT6 signaling could be upregulated under the profibrogenic environment, and bixin is able to inhibit IL4-induced STAT6 and ECM protein upregulation in HK2 cells.

### The Activation of STAT6 Signaling Is Essential for ECM Protein Expression

**FIGURE 3:**
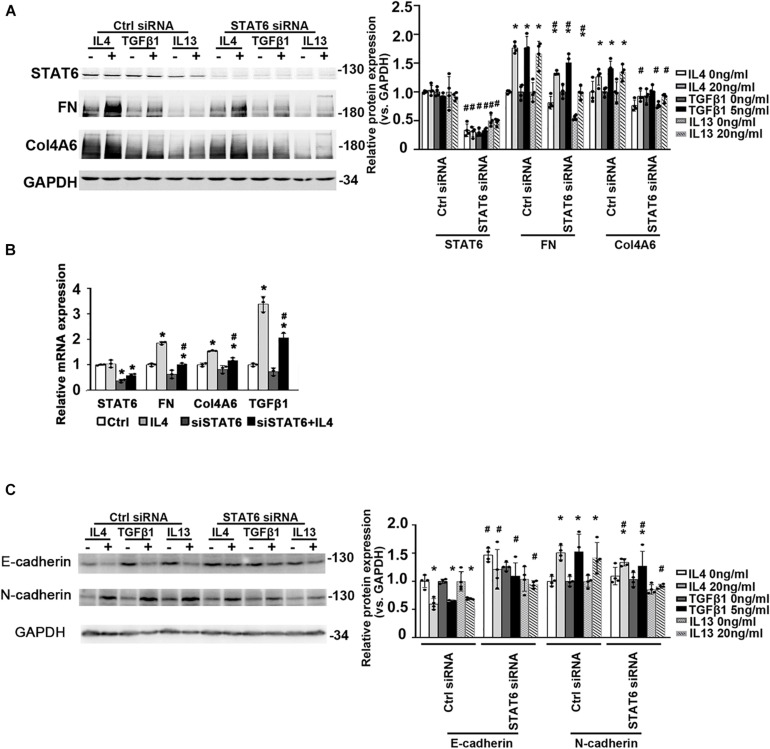
The activation of STAT6 signals is essential for ECM-related protein expression. **(A)** HK2 cells were transfected with either Ctrl siRNA or STAT6 siRNA. After 24-h serum free medium culture, the cells were treated with IL4 (20 ng/mL), TGFβ1 (5 ng/mL), or IL13 (20 ng/mL) for another 24 h. Cell lysates were harvested and subjected to immunoblot analysis with the indicated antibodies. Quantification of relative protein expression was determined. Results are expressed as means ± SD (*p < 0.05, Ctrl vs. treatments; ^#^*p* < 0.05, Ctrl siRNA transfection groups vs. STAT6 siRNA transfection groups; *n* = 4). **(B)** HK2 cells were transfected with either Ctrl siRNA or STAT6 siRNA for 24 h in serum free medium and followed by IL4 (20 ng/mL) treatment for another 24 h. The total RNA was extracted, and qRT-PCR assay was employed to measure the mRNA expression of STAT6, FN, Col4A6, and TGFβ1. Results are expressed as means ± SD (*p < 0.05, Ctrl vs. treatments; ^#^*p* < 0.05, Ctrl siRNA transfection groups vs. STAT6 siRNA transfection groups; *n* = 3). **(C)** HK2 cells were transfected with either Ctrl siRNA or STAT6 siRNA. After 24-h serum free medium culture, the cells were treated with IL4 (20 ng/mL), TGFβ1 (5 ng/mL), or IL13 (20 ng/mL) for another 24 h. Cell lysates were harvested and subjected to immunoblot analysis with the indicated antibodies. Quantification of relative protein expression was determined. Results are expressed as means ± SD (*p < 0.05, Ctrl vs. treatments; ^#^*p* < 0.05, Ctrl siRNA transfection groups vs. STAT6 siRNA transfection groups; *n* = 4).

To figure out if the suppression of STAT6 by bixin in renal tubular epithelium is necessary for renal interstitial fibrosis, we used HK2 cells. The ECM-related proteins (FN and Col4A6) were also increased with IL4, TGFβ1, and IL13 treatments as shown in Figure 3A, which could be suppressed by STAT6 knockdown. Consistently, the mRNA expression of FN, Col4A6, and TGFβ1 induced by IL4 treatment was downregulated when the cells were transfected with STAT6 siRNA, which demonstrated that a positive feedback loop may exist between the STAT6 signaling and TGFβ1 (Figure 3B). And E-cadherin was decreased by the administration of IL4, TGFβ1, and IL13, which could be reversed by STAT6 siRNA transfection. Consistently, the N-cadherin was increased with the profibrotic cytokines’ treatment and attenuated by STAT6 siRNA transfection as well (Figure 3C). These data indicated that the activation of STAT6 signaling in tubular epithelial cells was essential for the ECM deposition, and partial EMT seems to participate in this process.

### STAT6 Deficiency Decreases the TGFβ1 Expression and Prevents the Partial EMT of Renal Tubular Epithelium

**FIGURE 4:**
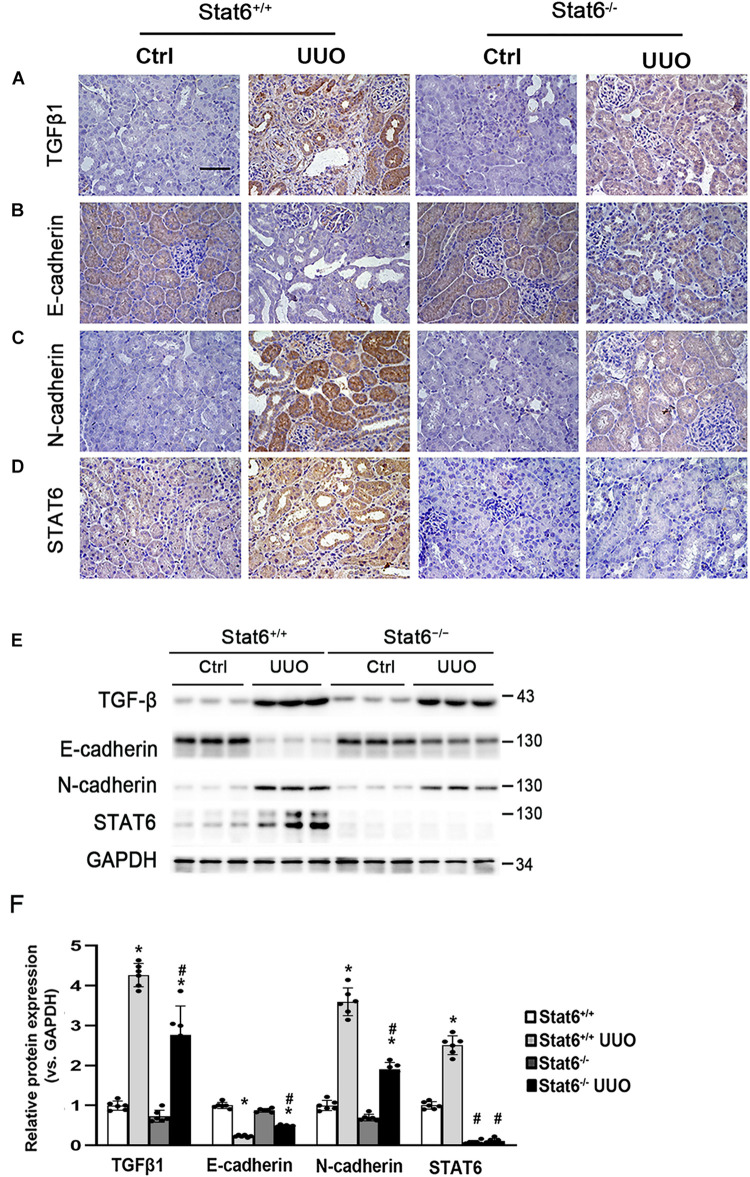
STAT6 deficiency decreases the TGFβ1 expression and prevents the partial EMT of renal tubular epithelium. Kidney sections of Stat6^+/+^ and Stat6^– /–^ mice with 7 days of UUO were subjected to IHC staining of TGFβ1 **(A)**, E-cadherin **(B)**, N-cadherin **(C)**, and STAT6 **(D)**; the representative images are shown (scale bar = 50 μm), **(E)** kidney tissue lysates from each group were subjected to immunoblot analyses with the TGFβ1, E-cadherin, N-cadherin, and STAT6. Representative blots of three independent samples in each group were shown; **(F)** quantification of relative protein expression was determined, results are expression as means ± SD (*p < 0.05, Ctrl vs. UUO group; ^#^*p* < 0.05, Stat6^+/+^ mice vs. Stat6^– /–^ mice; *n* = 6).

To determine whether STAT6 signaling is related with the partial EMT of the renal tubular epithelium and kidney fibrosis *in vivo*, the Stat6 wild-type (Stat6^+/+^) mice and knockout (Stat6^–/–^) mice with UUO were employed. After the mice were subjected with UUO for 7 days, the kidneys from the different groups were fixed and performed with IHC staining of TGFβ1 and EMT-related protein. As shown in Figure 4A, UUO induced a severe increase of TGFβ1 in the kidney of Stat6^+/+^ mice, but less TGFβ1 existed in Stat6^–/–^ UUO mice. Besides that, the EMT-related proteins E-cadherin and N-cadherin were stained as well for the assessment of partial EMT of renal tubular epithelium. UUO decreased the expression of E-cadherin and increased the N-cadherin in Stat6^+/+^ mice, which were restored in Stat6^–/–^ mice (Figures 4B,C). And as we expected, UUO could increase the expression of STAT6 in the renal tubular epithelium of Stat6^+/+^ mice, but without positive staining of STAT6 in the renal tubular epithelium of Stat6^–/–^ mice (Ctrl and UUO groups; Figure 4D). The results of the western blot analysis were consistent with the results of the IHC staining (Figure 4E). Taken together, these data indicated that STAT6 signaling is associated with the partial EMT of renal tubular epithelium, which would be the major effector cells of TGFβ1 signaling and lead to the progression of renal interstitial fibrosis.

### STAT6 Deficiency Improves Pathological Changes and Ameliorates the Renal Interstitial Fibrosis in the UUO Kidneys

**FIGURE 5:**
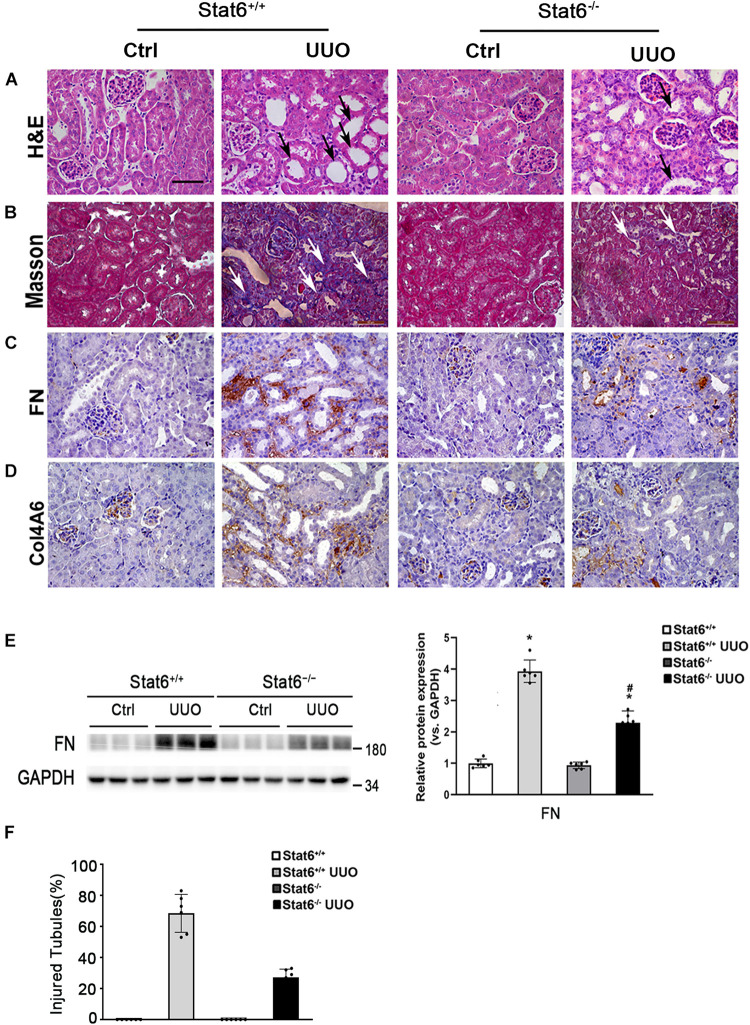
STAT6 deficiency ameliorates the renal fibrosis in the UUO kidney. Kidney sections of Stat6^+/+^ and Stat6^– /–^ mice with 7 days of UUO were subjected to H&E (black arrows: dilated and atrophic tubules) **(A)**, Masson (white arrows: collagen deposition) **(B)**, and IHC staining of FN **(C)**, and Col4A6 **(D)**; the representative images are shown (scale bar = 50 μm). **(E)** Kidney tissue lysates from each group were subjected to immunoblot analyses with the FN. Representative blots of three independent samples in each group were shown; quantification of relative protein expression was determined, results are expression as means ± SD (*p < 0.05, Ctrl vs. UUO group; ^#^*p* < 0.05, Stat6^+/+^ mice vs. Stat6^– /–^ mice; *n* = 6). **(F)** The graph showing the percentage of injured tubules for kidney tissues within groups (*p < 0.05, Ctrl vs. UUO group; ^#^*p* < 0.05, Stat6^+/+^ mice vs. Stat6^– /–^ mice; *n* = 6).

To further confirm whether STAT6 signaling regulates the renal fibrosis, the kidneys from the different groups were fixed and stained for the assessment of pathological changes and ECM deposition. As shown by H&E and Masson staining, UUO induced remarkable renal damage and interstitial ECM deposition in Stat6^+/+^ mice, which was attenuated in Stat6^–/–^ UUO mice (Figures 5A,B,F). The expression of FN and Col4A6 assessed by IHC staining was upregulated in renal tissues of Stat6^+/+^ UUO mice and was reserved obviously in the Stat6^–/–^ UUO mice (Figures 5C,D). Immunoblot analysis showed that STAT6 deficiency decreased the induced level of FN in the kidneys of the mice with UUO (Figure 5E). These data indicated that STAT6 deficiency improved pathological changes and ameliorated the renal interstitial fibrosis after UUO.

### Bixin Downregulates the Phosphorylation and Protein Stability of STAT6

**FIGURE 6:**
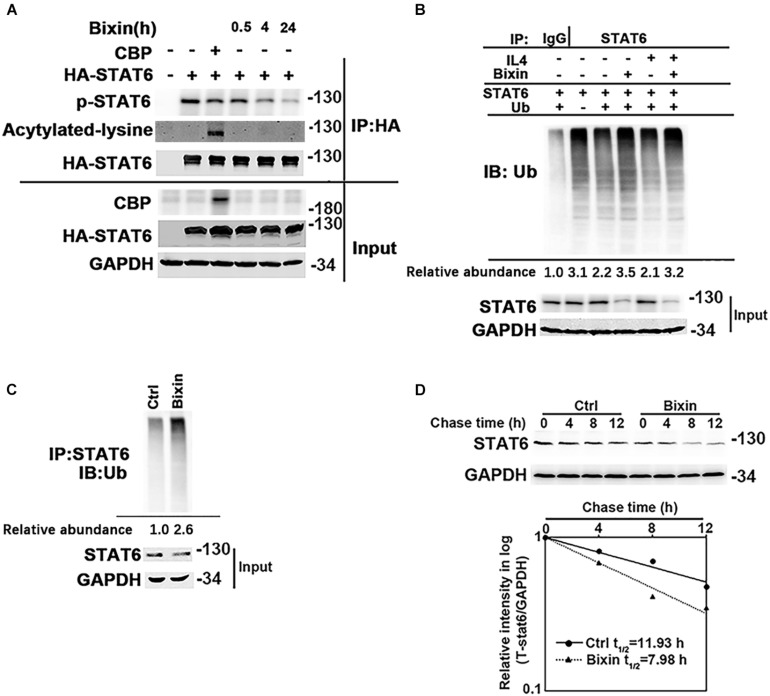
Bixin downregulates the phosphorylation and protein stability of STAT6. **(A)** HK2 cells were transfected with HA-STAT6 expression vector with or without CBP expression vector for 24 h in serum free medium. Cells were treated with bixin (40 μM) for the indicated time courses along with IL4 (20 ng/mL) for 24 h. The cell lysates were immunoprecipitated with HA-conjugated magnetic beads and analyzed by immunoblot analysis with anti–p-STAT6, anti–Pan-ack, and anti-HA antibodies. The total protein lysates was analyzed by immunoblot assay with anti-CBP, anti-HA, and anti-GAPDH antibodies (input). **(B)** HK2 cells were transfected with STAT6 expression vector along with or without ubiquitin expression vector for 24 h. Cells were treated with IL4 (20 ng/mL) and/or bixin (40 μM) for 24 h along with MG132 (10 μM) for 12 h. Anti-STAT6 immunoprecipitates were analyzed by immunoblot analysis with anti-Ub antibody for the detection of ubiquitin-conjugated STAT6. **(C)** HK2 cells were treated with bixin (40 μM) for 24 h along with MG132 (10 μM) for 12 h. Cell lysates were immunoprecipitated with an anti-STAT6 antibody and analyzed by immunoblot analyses with anti-ub antibody for the measurement of ubiquitin-conjugated STAT6. **(D)** HK2 cells were left untreated or treated with bixin (40 μM) for 24 h. CHX (50 μM) was added for the indicated time points, and cell lysates were subjected to immunoblot analyses with anti-STAT6 and anti-GAPDH antibodies. The intensity of STAT6 and GAPDH bands was quantified and plotted against the time after addition of CHX, and the half-life of STAT6 was determined.

Taken together, inhibition of STAT6 activity may mediate the protective effect of bixin for kidney interstitial fibrosis. The mechanisms of the suppression of STAT6 signaling caused by bixin treatment were further explored. According to previous studies, STAT6 can be degraded via ubiquitin-proteasome system and the lysosomal pathway (Gu et al., 2018). Bixin is recognized as an upstream regulator for P62, a major adaptor protein for autophagy degradation of ubiquitinated protein (Lin et al., 2013). First, the acetylation level of STAT6, which was negatively control the activation of STAT6 signaling, was determined. HK2 cells were transfected with the expression vectors of HA-tagged STAT6 and CBP. Cells were harvested, and an immunoprecipitation assay with HA-conjugated magnetic beads was performed. The results showed that bixin did not affect the acetylation levels of STAT6, but the phosphorylation of STAT6 was decreased in a time-dependent manner, which was consistent with Figure 1E. CBP transfection here was a positive control that showed the upregulation of the STAT6 acetylation (Figure 6A). Furthermore, the ubiquitination of STAT6 was investigated as well. After transfection with STAT6 and ubiquitin expression vectors, cells were treated with bixin and/or IL4 for 24 h. The ubiquitination of exogenous STAT6 at basal and induced levels was determined. Bixin treatment could upregulate the ubiquitination of STAT6 (Figure 6B). To further confirm the results, the ubiquitination of endogenous STAT6 was also detected as well. The results showed bixin could also increase the ubiquitination of endogenous STAT6 (Figure 6C). Because the changes of ubiquitination are usually associated with the protein degradation, the stability of STAT6 in the cells was next determined. HK2 cells were left untreated or treated with bixin for 24 h, and CHX (50 μM) was added for the indicated time points, and cell lysates were subjected to immunoblot analyses. The intensity of STAT6 and GAPDH bands was quantified and plotted against the time after addition of CHX. The results showed that the half-life of STAT6 with bixin treatment was decreased from 11.93 to 7.98 h (Figure 6D). Taken together, these data demonstrated that bixin could inhibit the STAT6 signaling by decreasing phosphorylation and stability of STAT6, which is related with the increase of STAT6 ubiquitination.

### Bixin Causes the STAT6 Protein Degradation by Proteasome and Autophagosome–Lysosome Systems

**FIGURE 7:**
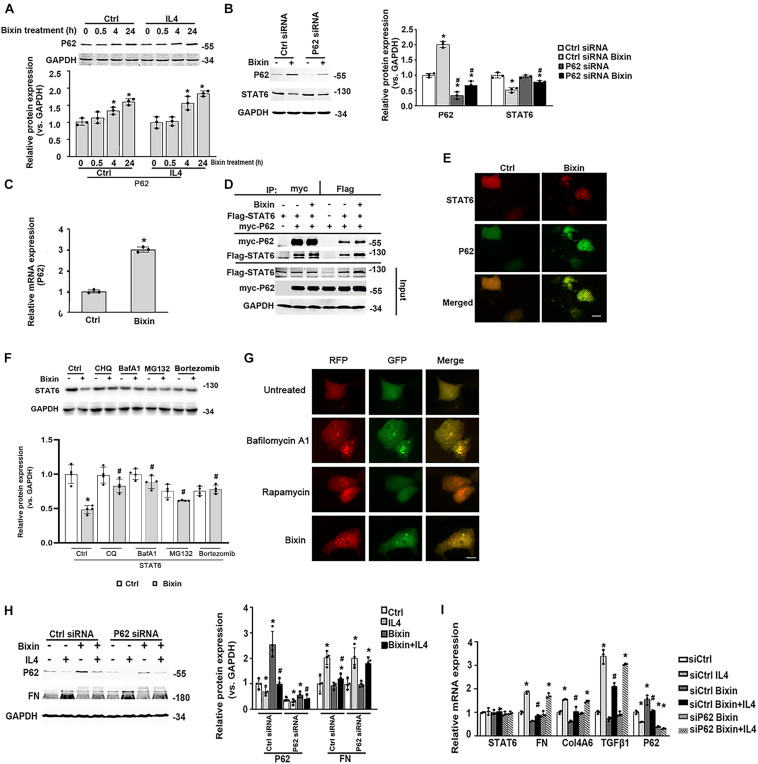
Bixin causes the STAT6 protein degradation by proteasome and autophagosome–lysosome systems. **(A)** After 24-h serum free medium treatment, HK2 cells were pretreated with IL4 (20 ng/mL) for 24 h. Followed by treatment with bixin (40 μM) for the indicated time courses, cells were lysed, and immunoblot analyses were employed for the detection of P62 expression. Results are expressed as means ± SD (*p < 0.05, Ctrl vs. bixin treatments; *n* = 3). **(B)** HK2 cells were transfected with Ctrl siRNA or P62 siRNA for 24 h in the serum free medium. Then the cells were left untreated or treated with bixin (40 μM) for 24 h. The cells were lysed and subjected to immunoblot analyses with the indicated antibodies. Quantification of the relative protein expression was determined. Results are expressed as means ± SD (*p < 0.05, Ctrl vs. bixin treatments; ^#^*p* < 0.05, Ctrl siRNA transfection group vs. P62 siRNA transfection group; *n* = 4). **(C)** HK2 cells were left untreated or treated with bixin (40 μM) for 24 h. The total RNA was extracted and analyzed by qRT-PCR assay. Results are expressed as means ± SD (*p < 0.05, Ctrl vs. bixin treatment; *n* = 3). **(D)** HK2 cells were transfected with the expression vector of Flag-tagged STAT6 and myc-tagged P62 for 24 h in serum free medium. The cell lysates were separately immunoprecipitated with Flag-conjugated agarose beads or myc-conjugated magnetic beads and analyzed by immunoblot analysis with anti-Flag, and anti-myc antibodies. The total protein was analyzed by immunoblot assay with anti-Flag, anti-myc, and anti-GAPDH antibodies (input). **(E)** HK2 cells were treated with bixin (40 μM) for 24 h. Cells were fixed and subjected to indirect immunofluorescence staining of STAT6 (red) and P62 (green); scale bar = 10 μm. **(F)** After culture with serum free medium for 24 h, HK2 cells were pretreated with CHQ (10 μM), BafA1 (100 nM), MG132 (10 μM) for 4 h, or bortezomib (100 nM) for 12 h. Then the cells were left untreated or treated with bixin (40 μM) for 24 h. The cell lysates were subjected to the immunoblot analyses with the indicated antibodies; quantification of relative protein expression was determined, and results are expressed as means ± SD (*p < 0.05, Ctrl vs. bixin treatments; ^#^*p* < 0.05, cells without pretreatment vs. cells with CHQ, BafA1, or MG132 pretreatment; *n* = 4). **(G)**. Kidney-cell imaging of HK2 cells transfected with a tandem mRFP-GFP-LC3 construct for 24 h and then either left untreated or separately treated with bafilomycin A1 (100 nM, 4 h), rapamycin (1 μM, 24 h), bixin (40 μM, 24 h); scale bar = 10 μm. **(H)** HK2 cells were transfected with either Ctrl siRNA or P62 siRNA for 24 h in serum free medium. After treatment with bixin (40 μM) and/or IL4 (20 ng/mL) for 24 h, cells were harvested, and the lysates were subjected to immunoblot analyses with the antibodies against P62, FN, and GAPDH; quantification of the relative protein expression was determined. Results are expressed as means ± SD (*p < 0.05, Ctrl vs. treatments; ^#^*p* < 0.05, IL4-treated group vs. bixin + IL4-treated group; *n* = 4). **(I)** HK2 cells were transfected with either Ctrl siRNA or P62 siRNA for 24 h in the serum free medium. Then cells were treated with bixin (40 μM) and/or IL4 (20 ng/mL) for 24 h. The total RNA was extracted, and the indicated mRNA expression was analyzed by qRT-PCR assay. Results are expressed as means ± SD (*p < 0.05, Ctrl vs. Treatments, ^#^*p* < 0.05, IL4 vs. bixin + IL4; *n* = 3).

Here, we further determined the expression of P62 in the cells with bixin treatment. Bixin could upregulate P62 expression in a time-dependent manner, which was not affected by IL4 treatment (Figure 7A). And P62 knockdown eliminated the STAT6 downregulation caused by bixin treatment (Figure 7B). Furthermore, bixin increased the P62 mRNA expression based on the qRT-PCR assay (Figure 7C). And the results from immunoprecipitation and immunofluorescence staining showed that P62 could directly bind with STAT6 in the cells, but bixin did not affect the affinity of binding between them (Figures 7D,E). Thus, these data demonstrated that bixin could increase the expression of P62, which could competitively bind with more STAT6 in the aggregates as shown in Figure 7E (yellow puncta in the cells).

To unveil the degradation of STAT6, the cells were pretreated with the inhibitors of autophagosome degradation (CHQ and BafA1) or proteasome degradation (MG132, bortezomib) for 4 h. Followed by treatment with bixin for another 24 h, cell lysates were subjected to immunoblot analyses. The results showed that the decrease of STAT6 caused by bixin was blocked by all of the inhibitors as shown (Figure 7F). To further clarify the effects of bixin on the process of autophagy, indirect immunofluorescence staining of LC3 showed that the expression of LC3 was up-regulated after treatment with bixin for 24 h in HK2 cells (Supplementary Figure 1). Moreover, a tandem mRFP–GFP–LC3 construct was employed, as previously reported, once the autophagosome fuses with the lysosome into the autophagolysosome, GFP fluorescence is quenched in this acidic environment. Nevertheless, mRFP is considerably more stable. Colocalization of GFP and RFP fluorescence (yellow puncta in the merged image) indicates that the fusion of autophagosome and lysosome is blocked, or the acidification of lysosome is abnormal, whereas RFP alone could be regarded as an autolysosome (Lau et al., 2013). Here, two positive controls were employed to differentiate increased formation of autophagosomes (rapamycin) from blockage of autophagosome degradation (bafilomycin A1). Cells treated with bafilomycin A1 contained yellow puncta (percentage of GFP and RFP fluorescence colocalization: 92.3%), but treatment with rapamycin had red puncta (percentage of GFP and RFP fluorescence colocalization: 2.5%). Interestingly, the red puncta were similarly observed in the cells treated with bixin (percentage of GFP and RFP fluorescence colocalization: 7.3%) (Figure 7G). Then, the effects of induced P62 caused by bixin on the IL4-induced fibrotic effects were tested. Similarly, bixin provided the antifibrotic effects on the suppression of FN induced by IL4. However, P62 siRNA transfection abated these effects; the mRNA levels of FN, Col4A6, and TGFβ1 confirmed these results. Moreover, bixin inhibited the activation of STAT6 signaling and was also reversed by P62 siRNA knockdown (Figures 7H,I). These results indicated that bixin could induce STAT6 degradation by proteasome and autophagosome–lysosome systems.

## Discussion

Through *in vitro* and *in vivo* studies, we showed for the first time that bixin, which is a widely used natural food colorant and additive, can promote STAT6 protein degradation in a proteasome and autophagosome–lysosome systems-dependent manner, and decreased STAT6 level in tubular cells inhibited partial EMT and ECM protein expression, which further alleviated UUO-caused kidney interstitial fibrosis.

We demonstrated that bixin could suppress the fibrotic effects by decreasing the expression of TGFβ1 and its targets (FN, Col4A6, and EMT-related proteins) with no observed cytotoxicity (Figures 1, 2E,F). STAT6 signaling was induced in the fibrotic kidneys after UUO, especially in the renal tubular epithelium. The upregulation of STAT6 was largely inhibited with bixin treatment under the profibrogenic environment (Figures 2A,D). To explore whether the downregulated STAT6 contributed to the protective effect for kidney fibrosis, we found the partial EMT process and renal interstitial fibrosis was attenuated in the kidney of STAT6 knockout mice after UUO (Figures 2–4). Even though we did not use the STAT6 renal tubular epithelial specific knockout mice in this work, STAT6 expression in the tubular epithelial cells was much stronger than other kidney cells in the kidney by IHC staining of STAT6 and p-STAT6 (Figures 2A, 4D). In addition, human renal tubular epithelial cell line HK2 was employed. *In vitro* studies showed that the activation of STAT6 signaling by profibrotic cytokines caused the EMT changes of renal tubular epithelial cells and the increase of ECM-related protein expression, which could be abated by silencing STAT6 expression (Figure 3). Taken together, our studies hinted that the partial EMT of tubular epithelial cells and renal interstitial fibrosis may be improved by bixin treatment through inhibiting the activation of STAT6 signaling in the renal tubular epithelium. Although whether EMT characterized by a complete phenotypic conversion actually contributes to kidney fibrosis is challenged, the viewpoint of “partial EMT” was validated to exist in tubular cells and is sufficient to promote kidney interstitial fibrosis (LeBleu et al., 2013; Grande et al., 2015; Lovisa et al., 2015; Zhou et al., 2017; Yuan et al., 2019).

Then we unveiled the mechanisms of the suppression of STAT6 signaling caused by bixin treatment. In detail, bixin could reduce the phosphorylation of STAT6 to negatively regulate the activation of STAT6 signaling (Figures 2B, 6A). According to previous studies, the activation of STAT6 signaling depends on the levels of its tyrosine phosphorylation and lysine acetylation (Goenka and Kaplan, 2011; Abroun et al., 2015). Once phosphorylated by JAK3, STAT6 could translocate into the nucleus and induce its targets by binding with the respective DNA promoter sites. Because DNA is negatively charged because of its phosphate backbone, and positively charged lysine in the DNA-binding domain of STAT6 stabilizes its association with a specific DNA sequence. While acetylation removes the positive charge of the lysine side chain from the transcription factor and thus inhibits its DNA-binding ability (Matsuzaki et al., 2005; Ren et al., 2016; Yu et al., 2019). However, bixin did not affect the lysine acetylation of STAT6 in the HK2 cells as shown in Figure 6A.

Additionally, we found that bixin also downregulated the stability of STAT6 by increasing its ubiquitination and decreasing the half-life in the HK2 cells (Figures 6B–D). As the increase of protein ubiquitination is often associated with protein degradation through proteasome and autophagosome–lysosome systems (Zeng et al., 2006; Kocaturk and Gozuacik, 2018). Thus, proteasome inhibitors (MG132 and CHQ) and autophagosome inhibitors (BafA1 and bortezomib) were used to determine the degradation of STAT6 caused by bixin treatment. The results demonstrated that the ubiquitinated STAT6 could be degraded by proteasome and autophagosome–lysosome systems. This finding is consistent with that of the previous reports (Gu et al., 2018) (Figures 7F,G).

Autophagy is a conserved catabolic mechanism required for cellular homeostatic quality control and regeneration. It is also a cellular stress response mechanism. P62 is a classical autophagy-related adaptor protein, which could transfer the ubiquitinated protein to autophagosome by binding with LC3 through its LIR domain (Moscat and Diaz-Meco, 2009; Glick et al., 2010; Nezis and Stenmark, 2012; Katsuragi et al., 2015; Aparicio et al., 2019). Here we found that bixin transcriptionally increased the expression of P62, which is related to the bixin-caused downregulation of STAT6 and antifibrotic effects (Figures 7A–C,H–I). The immunoprecipitation assay showed the existence of a direct interaction between P62 and STAT6, but bixin did not affect the affinity of this binding (Figures 7D,E). Moreover, P62 could bind with STAT6 in the puncta after bixin treatment (Figure 7E). These data demonstrated that the upregulation of P62 caused by bixin could lead to binding with more STAT6, which could be transferred to autophagosome for degradation, even though the qRT-PCR assay showed that bixin could increase the mRNA expression of P62. However, how this induction happened still needs clarification. We previously reported that bixin could activate the Nrf2 signaling via Keap1, and other studies reported that Nrf2 could induce P62 mRNA expression (Tao et al., 2015, 2016; Fan et al., 2018; Zhang et al., 2018). Thus, we speculated that bixin maybe increase the P62 expression in a Nrf2-dependent manner, which can be verified in our next STAT6 study. In addition, we found a direct binding between P62 and STAT6, which should not be related with ubiquitination level, because bixin treatment does not affect this bind affinity (Figure 7D). Thus, the exact binding sites of P62 and STAT6 also need to be explored in our next study.

Interestingly, in our study, we found that the STAT6 targets TGFβ1 could also activate STAT6 signaling as shown in Figure 2B. These data demonstrated that a positive feedback loop may exist between STAT6 signaling and fibrotic cytokine TGFβ1. If it works as expected, the interruption of this regulation would partially block the process of fibrosis, which would be a new strategy for treating fibrosis disease. As we reported previously, Nrf2 induced by bixin could suppress the TGFβ1 signaling in lung fibrosis (Zhang et al., 2018). In the further study, we will further explore if this regulation is STAT6 dependent to extend bixin’s antifibrosis mechanism for clinical therapeutic application.

**FIGURE 8:**
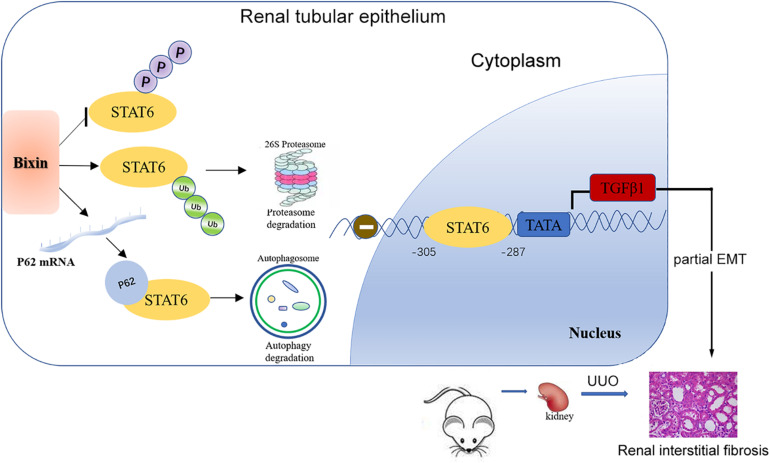
Mechanisms of the protective effect for kidney fibrosis treated with bixin. Bixin treatment could increase ubiquitination level of STAT6 and inhibit STAT6 phosphorylation in tubular cells. P62 mRNA level was also increased by bixin stimulation and facilitated ubiquitinated STAT6 degradation via proteasome and autophagosome–lysosome systems. TGFβ1, as a transcriptional target of STAT6, was suppressed and eventually attenuated renal partial EMT and interstitial fibrosis.

In summary, our study demonstrated that bixin treatment could improve partial EMT of tubular cells and renal interstitial fibrosis after UUO. The major mechanism of the protective effect of bixin occurs through the deactivation of STAT6 signaling by proteasome and autophagosome–lysosome systems (Figure 8).

## Data Availability Statement

The raw data supporting the conclusions of this article will be made available by the authors, without undue reservation.

## Ethics Statement

The animal study was reviewed and approved by Soochow University Institutional Animal Care and Use Committee.

## Author Contributions

ST, JL, LX, and HT designed the experiments. JL, YY, and SW conducted the experiments and co-wrote the manuscript. JL, YY, and LC analyzed the data. ST supervised the whole project and critically edited the manuscript text. All authors contributed to the article and approved the submitted version.

## Conflict of Interest

The authors declare that the research was conducted in the absence of any commercial or financial relationships that could be construed as a potential conflict of interest.

## Abbreviations

EMT: epithelial-to-mesenchymal transition
UUO: unilateral ureteral obstruction
CKD: chronic kidney disease
ECM: extracellular matrix
TGF β 1: transforming growth factor β 1
STAT: signal transducer and activator of transcription
JAK: Janus kinase
CBP: CREB-binding protein
CHX: cycloheximide
HRP: Horseradish peroxidase
DMEM: Dulbecco’s Modified Eagle Medium
siRNA: small interfering RNA
qRT-PCR: quantitative real-time polymerase chain reaction
SD: standard deviations
H&E: hematoxylin and eosin
IHC: immunohistochemistry
mRFP: mouse red fluorescent protein
NRF2: nuclear factor–erythroid 2 p45-related factor 2
FN: fibronectin.
