# Corrigendum: Bixin Protects Against Kidney Interstitial Fibrosis Through Promoting STAT6 Degradation

**DOI:** 10.3389/fcell.2020.643207

**Published:** 2021-01-29

**Authors:** Jianzhong Li, Youjing Yang, Shuhui Wei, Ling Chen, Lian Xue, Hailin Tian, Shasha Tao

**Affiliations:** ^1^Medical College of Soochow University, Suzhou, China; ^2^Department of Nephrology, The First Affiliated Hospital of Soochow University, Suzhou, China; ^3^School of Public Health, Medical College of Soochow University, Suzhou, China

**Keywords:** kidney fibrosis, bixin, STAT6, SQSTM1(P62), tubular cell

In the published article, there was an error regarding the affiliation for **Jianzhong Li and Shasha Tao**. As well as having affiliation(s) existed in the paper, they should also have ***Medical College of Soochow University, Suzhou, China*** as the first affiliation.

The authors apologize for this error and state that this does not change the scientific conclusions of the article in any way. The original article has been updated.

