# Supplementary material for: Bixin Protects Against Kidney Interstitial Fibrosis Through Promoting STAT6 Degradation
Source: Front Cell Dev Biol. 2020 Nov 17;8:576988. doi: 10.3389/fcell.2020.576988 (PMC7704619; doi:10.3389/fcell.2020.576988)
Supplement: Supplementary file 1 [file Image_1.PDF]

Supplemental Figure 1

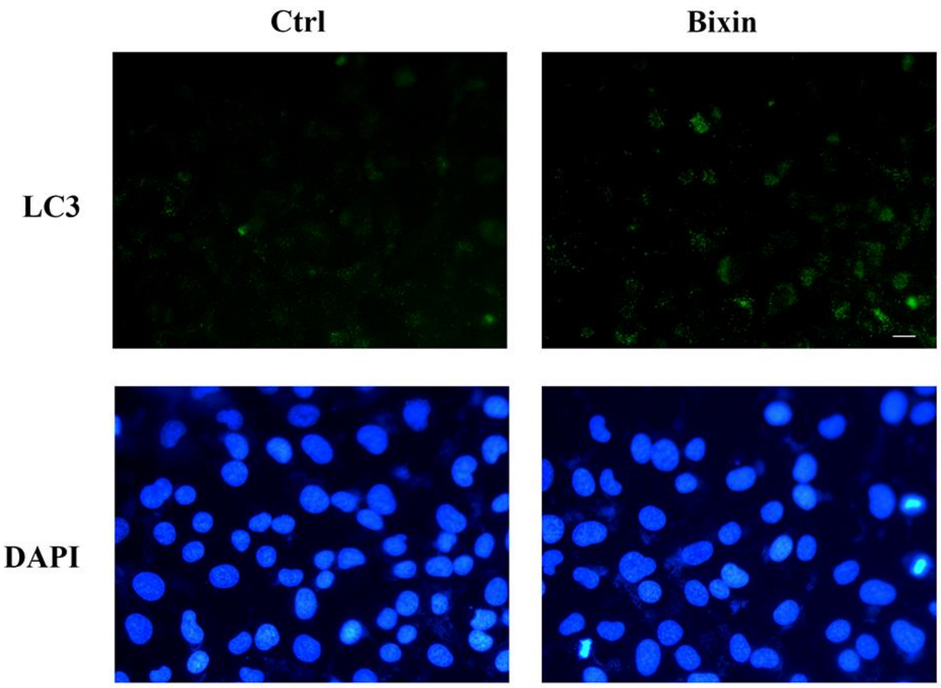

Supplemental Figure Legend

Supplemental Figure 1. Bixin induces the autophagy. (A) After treated with bixin (40 μM) for 24 h, HK2 cells were fixed and subjected to indirect immunofluorescence staining of LC3 (green); nucleus were stained with DAPI (the representative images were shown, scale bar = 100μm).
