# Supplementary figures and images for: Bixin Protects Against Kidney Interstitial Fibrosis Through Promoting STAT6 Degradation
Source: Front Cell Dev Biol. 2020 Nov 17;8:576988. doi: 10.3389/fcell.2020.576988 (PMC7704619; doi:10.3389/fcell.2020.576988)

Figure1D

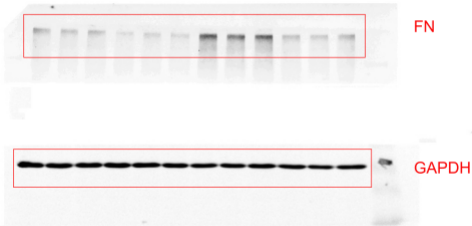

Supplement: Supplementary file 2 [file Data_Sheet_1.PDF]

Figure2B

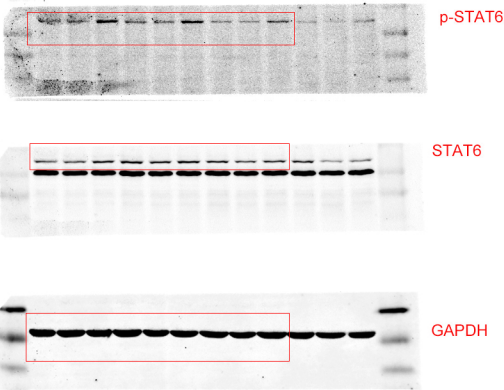

Figure2F

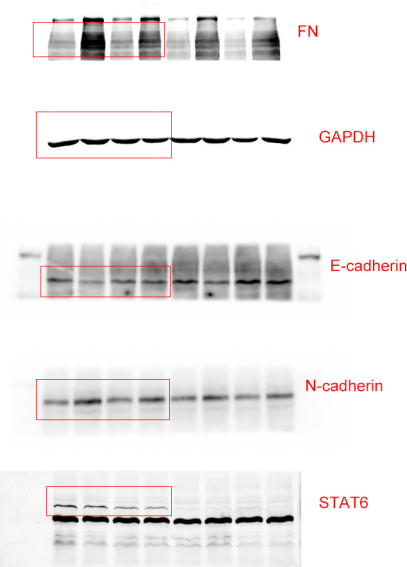

Figure2D

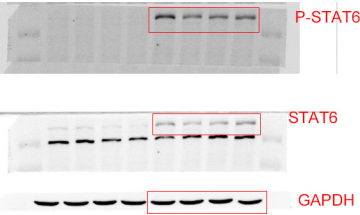

Supplement: Supplementary file 3 [file Data_Sheet_2.PDF]

Figure3A

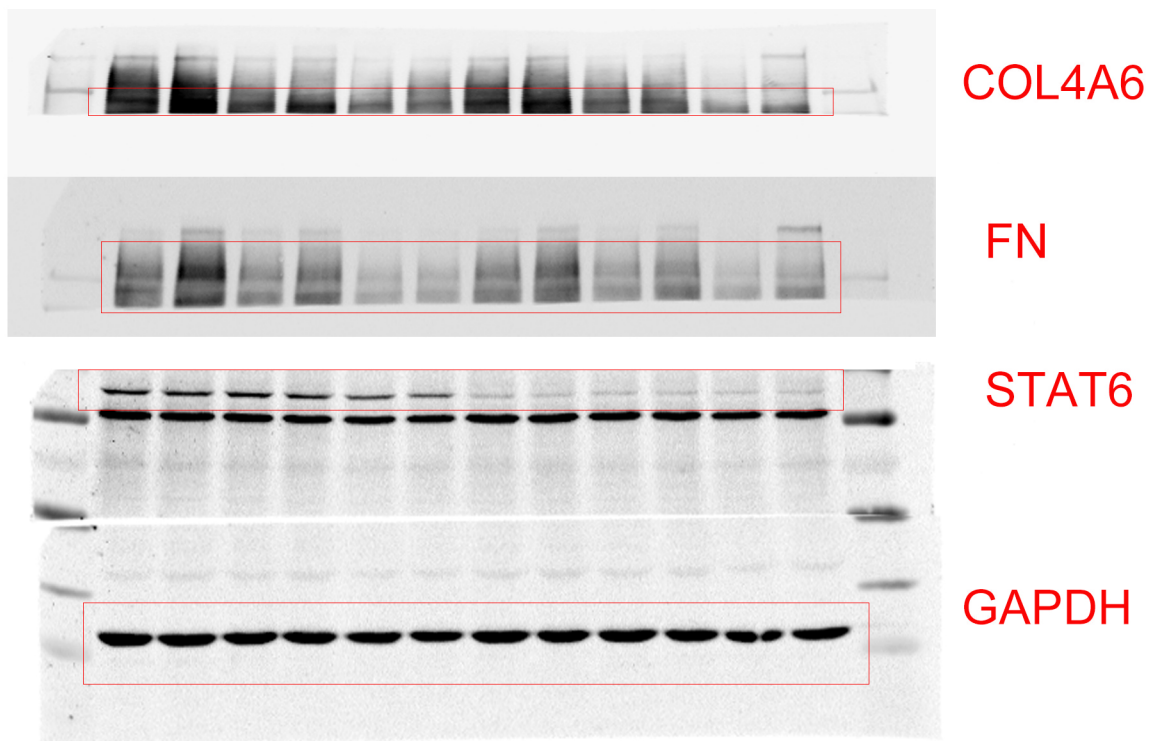

Figure3C

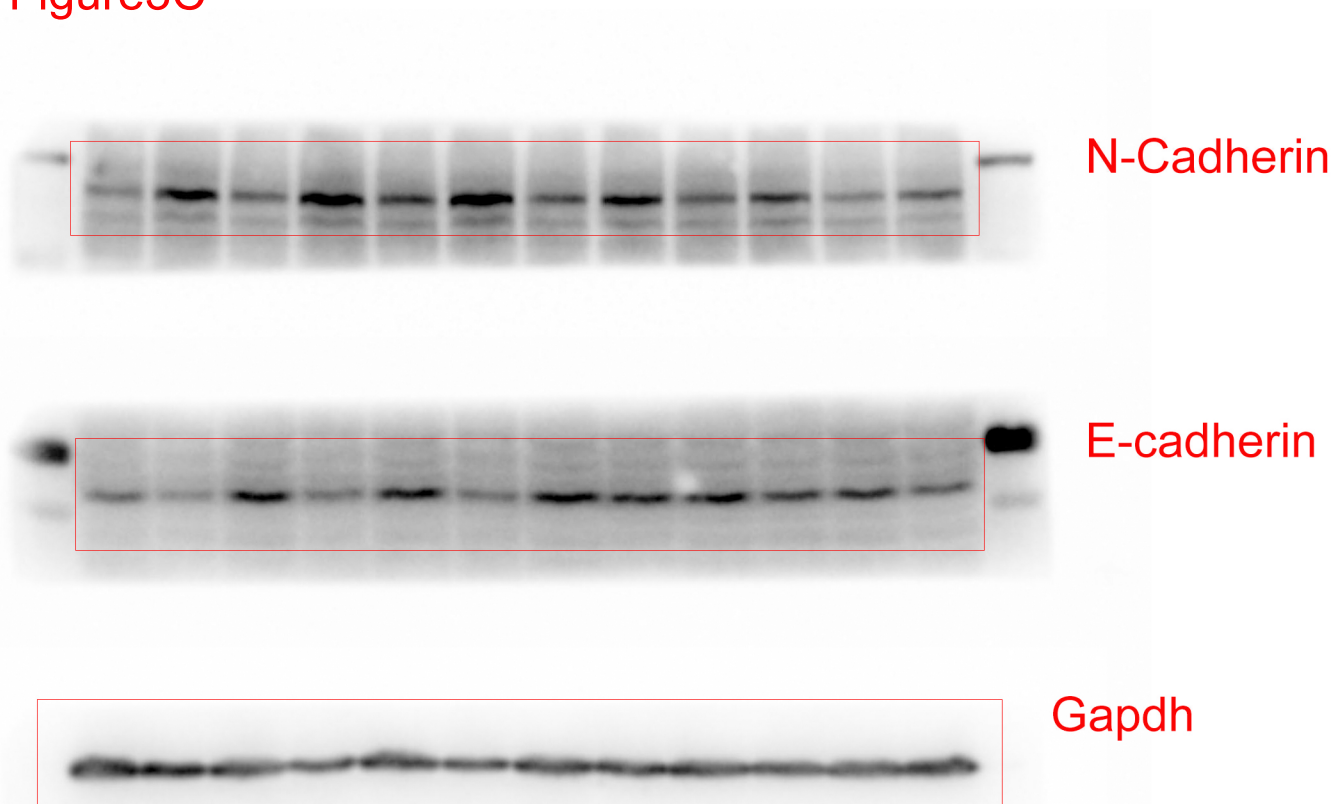

Supplement: Supplementary file 4 [file Data_Sheet_3.PDF]

Figure6A

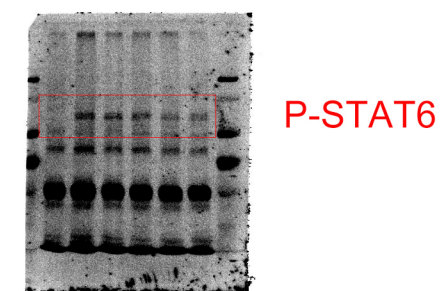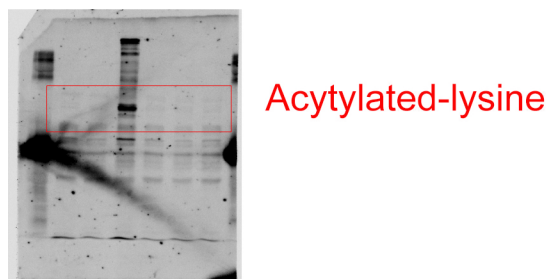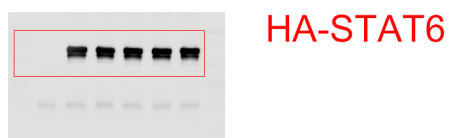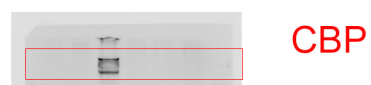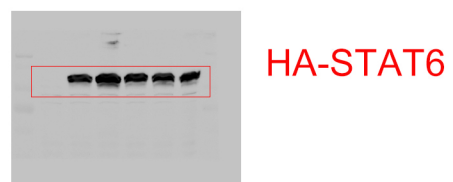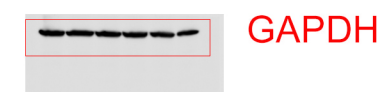

Figure6B

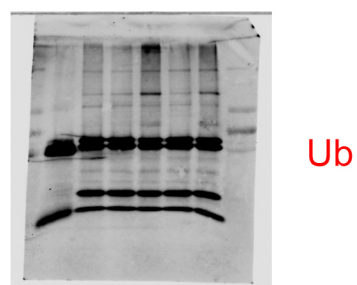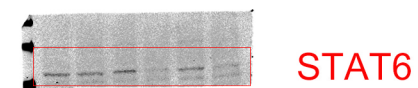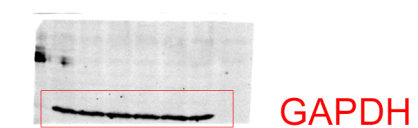

Figure6C

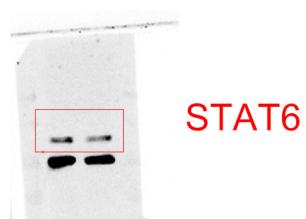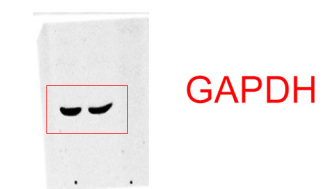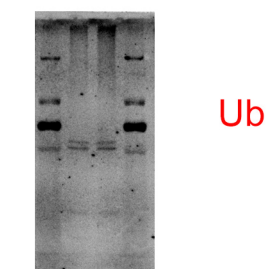

Figure6D

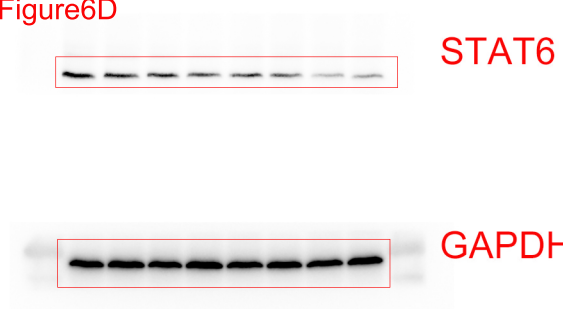

Supplement: Supplementary file 5 [file Data_Sheet_4.PDF]

Figure7A

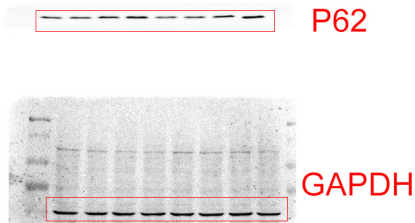

Figure7F

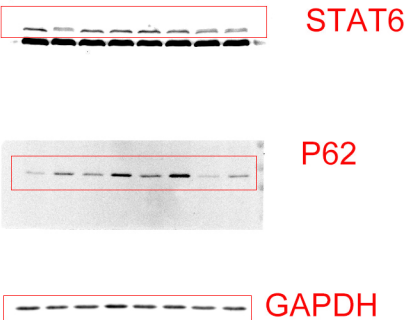

Figure7B

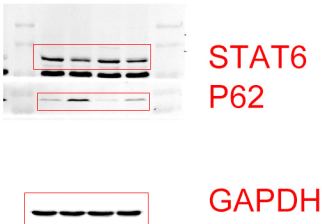

Figure7H

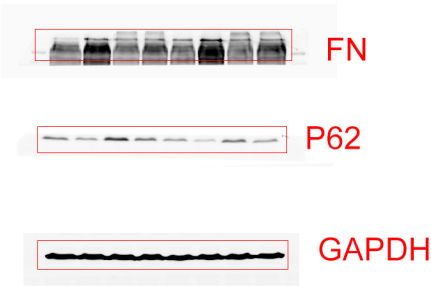

Figure7D

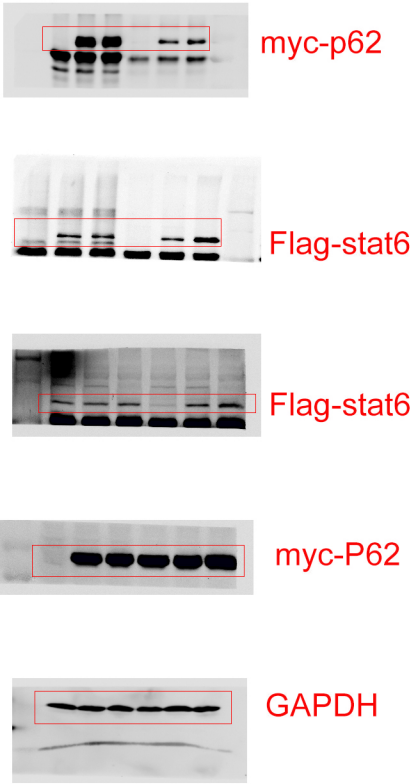

Supplement: Supplementary file 6 [file Data_Sheet_5.PDF]
